# The yeast 14-3-3 proteins Bmh1 and Bmh2 regulate key signaling pathways

**DOI:** 10.3389/fmolb.2024.1327014

**Published:** 2024-01-24

**Authors:** Veronika Obsilova, Tomas Obsil

**Affiliations:** ^1^ Institute of Physiology of the Czech Academy of Sciences, Laboratory of Structural Biology of Signaling Proteins, Division, BIOCEV, Vestec, Czechia; ^2^ Department of Physical and Macromolecular Chemistry, Faculty of Science, Charles University, Prague, Czechia

**Keywords:** 14-3-3 proteins, yeast, protein-protein interaction, phosphorylation, molecular mechanism, scaffolding, adaptor protein

## Abstract

Cell signaling regulates several physiological processes by receiving, processing, and transmitting signals between the extracellular and intracellular environments. In signal transduction, phosphorylation is a crucial effector as the most common posttranslational modification. Selectively recognizing specific phosphorylated motifs of target proteins and modulating their functions through binding interactions, the yeast 14-3-3 proteins Bmh1 and Bmh2 are involved in catabolite repression, carbon metabolism, endocytosis, and mitochondrial retrograde signaling, among other key cellular processes. These conserved scaffolding molecules also mediate crosstalk between ubiquitination and phosphorylation, the spatiotemporal control of meiosis, and the activity of ion transporters Trk1 and Nha1. In humans, deregulation of analogous processes triggers the development of serious diseases, such as diabetes, cancer, viral infections, microbial conditions and neuronal and age-related diseases. Accordingly, the aim of this review article is to provide a brief overview of the latest findings on the functions of yeast 14-3-3 proteins, focusing on their role in modulating the aforementioned processes.

## 1 Introduction

The 14-3-3 protein family includes highly conserved acidic proteins of approximately 30 kDa in size. These proteins are abundantly expressed in all eukaryotes and often in multiple isoforms. The yeast *Saccharomyces cerevisiae* expresses only two isoforms, Bmh1 and Bmh2. Encoded by the *BMH1* and *BMH2* genes, these isoforms are essential in most laboratory strains (van Heusden et al., 1992). Disrupting one of the *BMH* genes has a negligible effect on yeast cells, whereas simultaneous deleting both genes is lethal, clearly indicating that these isoforms have redundant but nonetheless crucial functions (van Heusden et al., 1995).

Bmh proteins commonly function as adaptors in signal transduction pathways by binding phosphorylated proteins, thereby activating, inactivating or sequestering them (van Heusden and Yde Steensma, 2006; van Heusden, 2009). Both Bmh proteins form stable dimers, similar to the 14-3-3 protein isoforms in higher eukaryotes (Yaffe et al., 1997), with a clear preference for heterodimer formation (Chaudhri et al., 2003). A thorough proteomic analysis using multistep immunoaffinity purification and mass spectrometry has identified 271 yeast proteins that specifically interact with Bmh proteins in a phosphorylation-dependent manner (Kakiuchi et al., 2007). Phosphorylation-dependent interactions with Bmh proteins underlie a wide range of physiological roles (Obsilova and Obsil, 2022). Since protein phosphorylation regulates both cell cycle and metabolism, the analysis of the budding yeast cell cycle phosphoproteome has unsurprisingly revealed more than 200 sites on metabolic enzymes and transporters regulated by phosphorylation (Zhang et al., 2019). Among the thousands of sites whose phosphorylation increased during the cell cycle, both Cdk-phosphorylated and R-R-X-S motifs were highly enriched. Moreover, such motifs are phosphorylated by PKA and recognized by 14-3-3 proteins (Zhang et al., 2019). These data suggest that binding interactions with Bmh proteins participate in a number of key cellular processes.

The aim of this review is to provide an overview of the latest findings on the functions of yeast 14-3-3 proteins. Notwithstanding our efforts to update readers on the state of the art on the role of Bmh proteins in key yeast processes, they should also refer to excellent reviews on Bmh proteins previously published since the turn of the century (van Hemert et al., 2001; van Heusden, 2005; van Heusden and Yde Steensma, 2006; van Heusden, 2009; Conrad et al., 2014; Kumar, 2017).

## 2 Yeast 14-3-3 proteins

Yeasts, unlike higher eukaryotes, usually express only one or two 14-3-3 protein isoforms. In the yeast *S. cerevisiae*, these isoforms are encoded by the *BMH1* and *BMH2* genes. In 1991, the gene for the Bmh1 protein was discovered by chance when the neighboring PDA1 gene was cloned, and this newly discovered protein was named brain modulosignaling homologue (Bmh) for its similarity to 14-3-3 proteins previously characterized in brain tissue and known to specifically modulate other proteins (van Heusden et al., 1992). Three years later, the second gene, *BMH2*, was identified by sequence homology (van Heusden et al., 1995; van Heusden, 2005).

Both Bmh proteins are interchangeable because deleting either gene has little effect on yeast cell viability, growth, sporulation efficiency, or even spore viability. However, simultaneous disrupting both *BMH* genes is lethal in most strains, suggesting that 14-3-3 proteins play key roles in yeast cellular processes (van Heusden et al., 1992; van Heusden and Yde Steensma, 2006). Further demonstrating that Bmh proteins have crucial functions in yeast, *BMH1* is not required for yeast growth in rich medium, but deleting both *BMH* genes causes severe growth defects and increases sensitivity to various stressors (Kumar and Srivastava, 2016; Kumar, 2017).

As for other yeast 14-3-3 proteins, *Schizosaccharomyces pombe* also express two genes encoding 14-3-3 proteins. These proteins were named *rad24* and *rad25* because they were isolated for their association with radiation sensitivity (Ford et al., 1994). Similarly, the dimorphic yeast *Yarrowia lipolytica* also contains two 14-3-3 genes, *YlBMH1* and *YlBMH2* (Hurtado and Rachubinski, 2002). Conversely, the pathogenic yeast *Candida albicans* thrives despite expressing only one 14-3-3 gene (Cognetti et al., 2002). All yeast 14-3-3 proteins have nevertheless remained highly conserved during evolution, as did 14-3-3 isoforms of higher eukaryotes, albeit with some variability. This variability is persistently found in loops connecting individual α-helices and especially in the C-terminal stretch.

**FIGURE 1:**
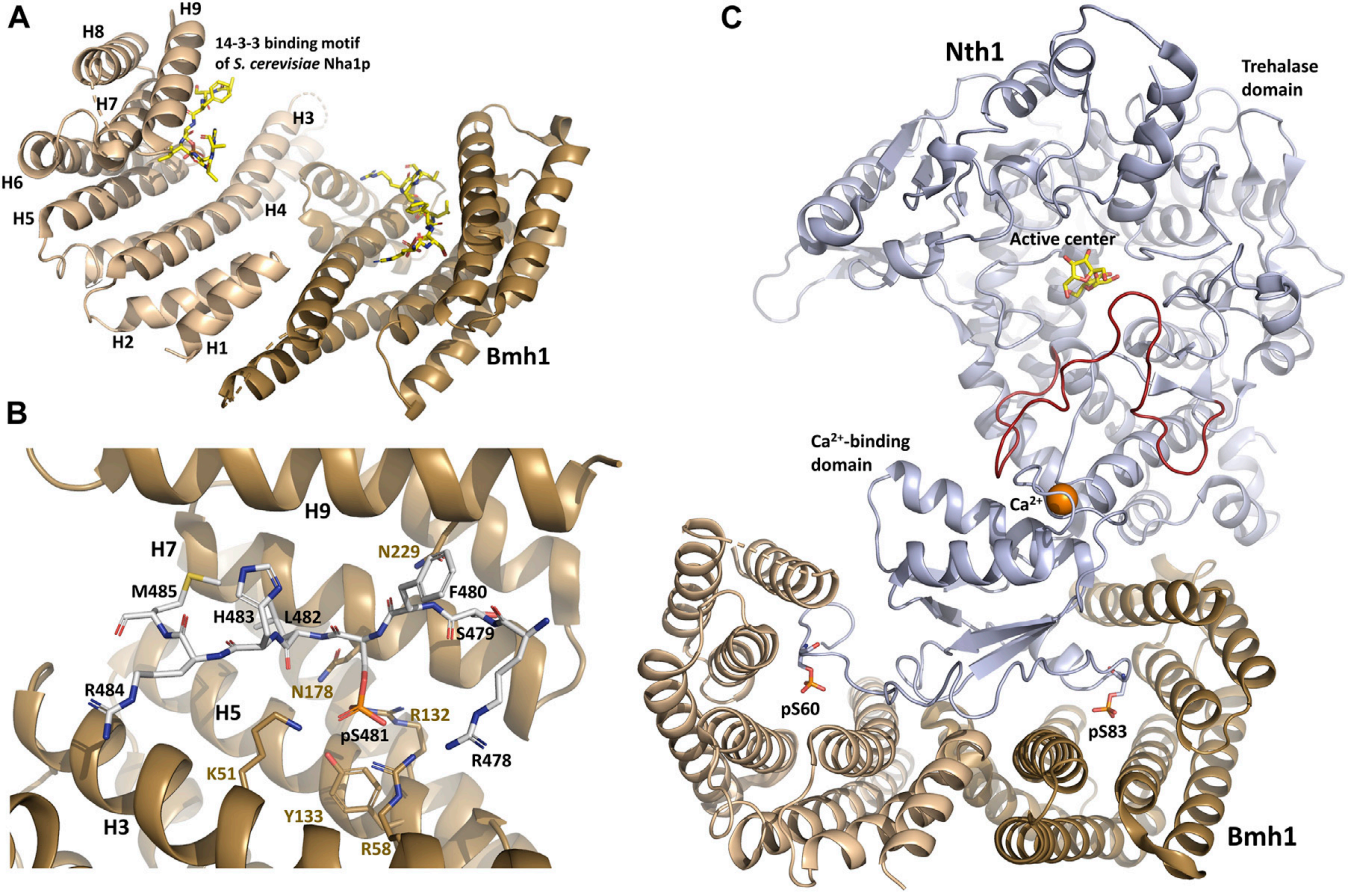
Structure of the yeast Bmh1 protein. **(A)** Crystal structure of the *Saccharomyces cerevisiae* Nha1p (Na^+^/H^+^ antiporter) 14-3-3 binding motif Ser^481^ bound to Bmh1 dimer (PDB ID: 6QK8) (Smidova et al., 2019). **(B)** Detailed view of interactions between the ligand binding groove of Bmh1 and the 14-3-3 binding motif Ser^481^ of *Saccharomyces cerevisiae* Nha1p (RSFpS^481^LHRM). **(C)** Crystal structure of the *Saccharomyces cerevisiae* Nth1:Bmh1 complex (PDB ID: 5N6N) (Alblova et al., 2017). The flexible part of the catalytic trehalase domain that is stabilized by the formation of the complex is shown in red. The figure was prepared with PyMOL (https://pymol.org/2/).

All 14-3-3 isoforms, including Bmh1/2 proteins, recognize three canonical phosphorylated motifs, namely R[S/Φ][+]pSXP (mode I) (Yaffe et al., 1997), RX[Φ/S][+]pSXP (mode II) (Rittinger et al., 1999) and pS/pT-X_1–2_-COOH (C-terminal mode III) (Ganguly et al., 2005), where pS is phosphoserine, Φ is an aromatic residue, + is a basic residue, and X is any type of residue (usually Leu, Glu, Ala, and Met). In addition to these canonical motifs, 14-3-3 proteins can also bind to motifs with considerably divergent features, including non-phosphorylated moieties (reviewed in (Obsilova and Obsil, 2022)). As shown by structural studies, 14-3-3 proteins form highly helical dimers, with each protomer consisting of nine antiparallel α-helices (H1-H9) (Eisenreichova et al., 2016; Alblova et al., 2017; Smidova et al., 2019). The shape of the 14-3-3 dimer molecule resembles that of a cup with approximate dimensions of 35 Å × 35 Å × 20 Å (width, length and depth) (Figure 1A). The 14-3-3 dimer can simultaneously bind to two phosphorylated motifs located either within the same target protein or in two different proteins thanks to two amphiphilic grooves formed by the α-helices H3, H5, H7 and H9 (Liu et al., 1995; Xiao et al., 1995; Obsil et al., 2001; Obsil et al., 2003; Veisova et al., 2012).

When bound to various 14-3-3 isoforms, short synthetic phosphopeptides adopt an extended conformation with a fixed orientation in the ligand binding groove of 14-3-3, without inducing substantial structural changes in the 14–3-3 dimer (Yaffe et al., 1997; Rittinger et al., 1999; Yang et al., 2006; Ottmann et al., 2007; Sluchanko and Gusev, 2017; Psenakova et al., 2018; Kalabova et al., 2020; Horvath et al., 2021; Pohl et al., 2021). The phosphate group of pSer/pThr binds to a positively charged pocket within the amphipatic ligand-binding groove of 14-3-3 formed by conserved residues Lys^51^, Arg^58^, Arg^132^ and Tyr^133^ (Bmh1 numbering) located in helices H3 and H5 (Figure 1B). In addition, the Pro residue often found at position +2 with respect to pSer/pThr in 14-3-3 ligand-binding motifs creates a kink in the polypeptide chain. This abrupt change in the direction of the polypeptide chain enables its exit from the binding groove, thereby enhancing binding affinity and stabilizing 14-3-3 protein-ligand interactions (Yaffe et al., 1997; Rittinger et al., 1999).

Although Bmh1 and Bmh2 show a high degree of homology to 14-3-3 proteins of other organisms, they differ from the isoforms of higher eukaryotes in the sequence and length of their C-terminal segment (van Heusden and Yde Steensma, 2006; van Heusden, 2009). The C-terminal segment of Bmh proteins is considerably longer and contains a polyglutamine repeat with an unknown function. Bmh1 contains a stretch of 10 consecutive glutamines, whereas Bmh2 contains as many as 17 consecutive glutamines. Regardless of their length, these polyQ stretches have prevailed during evolution and may be related to the ability to interact with prion-like domains, as previously shown for other polyQ-containing proteins (Ripaud et al., 2014; Gerbich and Gladfelter, 2021). These features prevent the C-terminal segment from interacting with the ligand binding groove and functioning as an auto-inhibitor, as described for some mammalian isoforms (Truong et al., 2002; Obsilova et al., 2004; Silhan et al., 2004; Veisova et al., 2010).

## 3 14-3-3 proteins regulate fundamental cellular processes in yeast

14-3-3 proteins regulate their binding partners through several mechanisms, including conformational modulation of the ligand. When the protein bound to 14-3-3 is an enzyme, this interaction can modulate its enzymatic activity. Such modulation is exemplified by the activation of the yeast neutral trehalase Nth1, as discussed in Section 3.2. 14-3-3 binding can also mask the binding site(s) of other proteins or binding partners, such as an RNA-binding site, in regulating Rim4 during meiosis (Zhang et al., 2023), as discussed in Section 3.3. And in yet another mechanism, 14-3-3 proteins regulate the subcellular localization of their binding partners, as in the regulation of the mitochondrial retrograde (RTG) signaling pathway (Rios-Anjos et al., 2017) and the stress-responsive transcriptional activators Msn2 and Msn4 (Beck and Hall, 1999), which are discussed below, in Sections 3.6 and 3.7, respectively.

### 3.1 14-3-3 proteins are key players in catabolite repression

Glucose repression (also known as catabolite repression) is an essential mechanism for budding yeast whereby high levels of glucose and fructose, the preferred carbon sources, repress the expression of enzymes required for the catabolism of other carbon sources (Gancedo, 1998). The key component of the glucose repression pathway is the protein kinase Snf1, the yeast homolog of AMP-activated protein kinase (AMPK) (Carlson, 1999). Snf1 is inhibited by glucose and activated in its absence by phosphorylation of Thr^210^ in the activation loop (Rubenstein et al., 2008). This phosphorylation is promoted by the activity of three partly redundant upstream kinases, namely Sak1, Tos3 and Elm1, and counteracted by protein phosphatase 1 (PP1) comprising the regulatory subunit Reg1 and the catalytic subunit Glc7 (Conrad et al., 2014). Activated Snf1 stimulates transcription of genes needed for growth on nonglucose carbon sources by phosphorylating Mig1 (a zinc finger and DNA binding protein that mediates glucose repression), thereby promoting its transport from the nucleus to the cytoplasm (De Vit et al., 1997). However, more recent studies have shown that Mig1 constantly shuttles between the nucleus and cytoplasm regardless of the glucose presence and Snf1 activation (Bendrioua et al., 2014; Wollman et al., 2017). Snf1 also controls the transcription of glucose-repressed genes, such as transcription factors Cat8 and Adr1, which subsequently control the expression of hundreds of glucose-repressed genes through direct binding and transcription activation (Bruckmann et al., 2004; Ichimura et al., 2004; Braun et al., 2014). Adr1 activity is regulated at multiple levels and affected by Bmh binding (Young et al., 2012).

**FIGURE 2:**
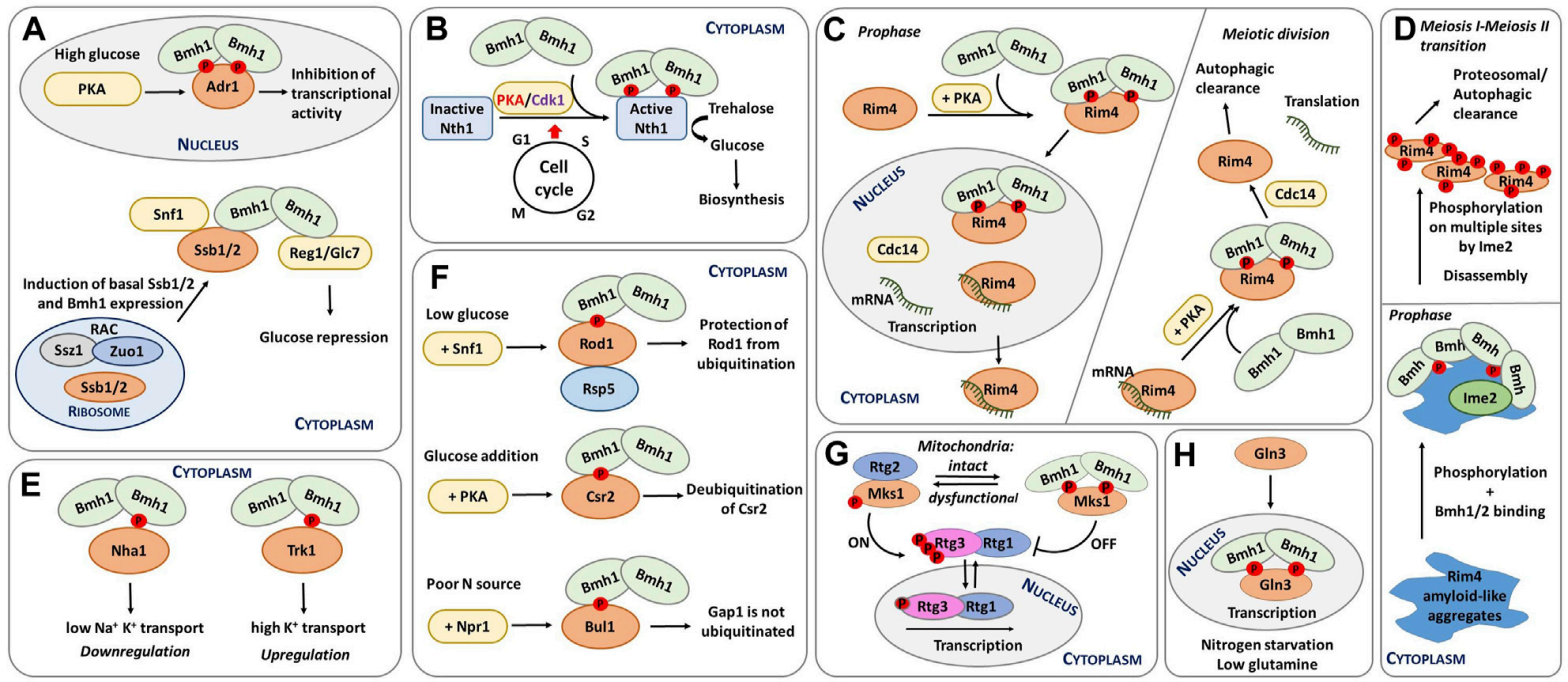
Schematic representation of selected functions of yeast 14-3-3 proteins. **(A)** Transcriptional regulation of glucose-repressed genes. Under high-glucose conditions, Adr1 is phosphorylated by PKA, which leads to Bmh binding. The formation of this complex inactivates Adr1 (Braun et al., 2013). In addition, Ssb1/2 and Bmh1 regulate Snf1, with Ssb directly interacting with Bmh. The resulting complex functions as a chaperone module of Snf1 (Hubscher et al., 2016; Hubscher et al., 2017). Furthermore, RAC is also regulates Snf1 activity by increasing Ssb1/2 and Bmh1/2 mRNA levels during growth on glucose (Hubscher et al., 2016; Yamada et al., 2023). **(B)** Regulation of neutral trehalase Nth1. Nth1 activity is regulated by the coordinated action of PKA, Cdk1 and Bmh during the cell cycle transition from G1 to S phase. Phosphorylation and Bmh binding activate Nth1, which subsequently converts trehalose into glucose for further biosynthetic events (Veisova et al., 2012; Dengler et al., 2021). **(C)** Spatiotemporal control of meiosis by 14-3-3 proteins. Left: before meiotic divisions, Cdc14 is upregulated; as a result, the Rim4-mRNA complex is transferred to the nucleus, and the Rim4-Bmh1/2 complex breaks down. Right: During meiotic anaphase, increased PKA activity releases Rim4 from mRNA, thus forming the Rim4-Bmh1/2 complex. In the cytoplasm, Cdc14 induces the disassembly of the Rim4-Bmh1/2 complex, Rim4 degradation, and translation of Rim4-sequestered mRNAs. Modified from (Herod et al., 2022; Zhang et al., 2023). **(D)** Bmh participate in the phosphorylation-mediated clearance of Rim4 aggregates during meiosis, thereby contributing to global protein aggregate homeostasis. Bmh proteins bind to Rim4 amyloid-like aggregates and facilitate Rim4 phosphorylation by the meiosis-specific kinase Ime2. This phosphorylation subsequently leads to the disassembly of Rim4 aggregates. Modified from (Herod et al., 2022). **(E)** Different mechanisms of regulation of yeast transporters by 14-3-3 proteins. 14-3-3 binding downregulates the cation efflux activity of Nha1 but upregulates the activity of the potassium transporter Trk1 (Smidova et al., 2019; Masaryk et al., 2023). **(F)** Endocytosis regulation. Under low glucose conditions, Rod1 is phosphorylated by Snf1 and inactivated by the formation of a complex with 14-3-3 proteins. This complex protects Rod1 from ubiquitination (Becuwe et al., 2012b). Adding glucose induces Csr2 phosphorylation by PKA and subsequent 14-3-3 protein binding, keeping Csr2 deubiquitylated (Hovsepian et al., 2017). Under low nitrogen levels, the Npr1 kinase phosphorylates the Bul adaptors, which are inhibited by 14-3-3 protein binding. 14-3-3 protein binding, in turn, prevents Gap1 ubiquitylation, maintaining this general amino acid permease active at the plasma membrane (Merhi and Andre, 2012). **(G)** Dynamic regulation of mitochondrial retrograde signaling. When cells have functional mitochondria, the RTG pathway is inactive, phosphorylated Rtg3 is bound to Rtg1 in the cytoplasm, and phosphorylated Mks1 is bound to 14-3-3. Dysfunctional mitochondria trigger the activation of the RTG pathway. Under these conditions, Mks1 is dephosphorylated, binding to Rtg2 and, thus, prompting the translocation of the Rtg1-Rtg3 complex into the nucleus and the transcription of genes involved in the retrograde response. Adapted from (Bui and Labedzka-Dmoch, 2023). **(H)** Regulation of rapamycin-mediated cell signaling and nitrogen catabolite repression. Upon rapamycin treatment, Gat1 and Gln3 translocate into the nucleus and activate the transcription of target genes. Upon nitrogen starvation and low glutamine, Gln3 remains in the nucleus bound to Bmh and mediates NCR-sensitive transcription (Tate et al., 2017).

The involvement of Bmh proteins in glucose repression was first revealed by their interaction with Reg1 (Mayordomo et al., 2003; Dombek et al., 2004). More specifically, Bmh proteins bind to the conserved 187-232 region within the N-terminal part of phosphorylated Reg1. This interaction is required to maintain complete repression, but Bmh proteins also function independently of Reg1 during glucose repression. As a case in point, simultaneous deletion of both genes encoding these proteins causes synergistic release from suppression, at least partly due to direct Bmh binding to the phosphorylated regulatory domain of promoter-bound transcription factor Adr1 (Figure 2A) (Braun et al., 2013).

Adr1 is a nutrient-regulated transcription factor that coactivates genes needed for growth in the absence of a fermentable carbon source. Under high-glucose conditions, Adr1 is phosphorylated by PKA, which enables Bmh binding to Adr1, preventing transcriptional activation by Adr1. Under low glucose conditions, Adr1 is activated by dephosphorylation by the protein phosphatase complex Glc7-Reg1 in a Snf1-dependent manner. In short, Bmh inhibits Adr1 at high glucose concentrations, and the Glc7-Reg1 complex overrides Adr1 repression at low glucose concentrations. Snf1 may phosphorylate and consequently activate Adr1 by phosphorylating a site or sites in the DNA-binding domain of Adr1 (Young et al., 2008; Ratnakumar et al., 2009), whereas PKA inactivates Adr1 by phosphorylating sites C-terminal to the DNA-binding domain, particularly Ser^230^, thus allowing Bmh binding. So, Bmh controls the transcriptional activation by Adr1 by directly binding to PKA-dependent phosphorylation sites, most likely modulating the ability of Adr1 to activate transcription (Parua et al., 2010; Parua and Young, 2014).

Another adaptor protein crucial for glucose repression through its interaction with Bmh is the yeast Hsp70 homolog Ssb1 (Dombek et al., 2004). As a chaperone, Ssb1 directly interacts with unfolded polypeptide substrates while residing on the ribosome, but half of the Ssb molecules remain in the cytoplasm (Peisker et al., 2010). In turn, cytosolic Ssb1/2 is associated with the Snf/Glc7 signaling pathway as a member of the Snf1 complex, which also contains Reg1 (Elbing et al., 2006). Ssb1/2 and Bmh1 post-translationally regulate Snf1, with Ssb directly interacting with Bmh and the resulting complex functioning as a novel chaperone module that modulates Snf1 (Hubscher et al., 2016; Hubscher et al., 2017).

The conserved yeast ribosome-associated complex (RAC) also regulates Snf1 activity (Hubscher et al., 2016; Zhang et al., 2017). This multiprotein complex is formed by Ssz1 (non-canonical Hsp70) and Zuo1 (J-protein). Zuo1 is also involved in glucose repression, possibly by increasing Ssb1/2 and Bmh1/2 mRNA levels during growth on glucose (Yamada et al., 2023). However, the exact mechanism whereby RAC and Ssb collaborate with Bmh to modulate Snf1 under various physiological conditions remains unknown. Further structural research must be conducted to elucidate the details of these interactions and their functional significance.

### 3.2 14-3-3 proteins regulate trehalase activity and carbon metabolism

Yeast metabolism, growth and division must adapt to available nutrients. This adaption mechanism is mediated by cross-talk between nutrient signaling, metabolism, growth and the cell cycle (Ewald, 2018). In the G1 phase, excess nutrients can be stored as trehalose and glycogen. These storage carbohydrates are then used for biosynthetic processes in the S, G2 and M phases of the cell cycle (Figure 2B) (Crowe et al., 1984). Intracellular trehalose levels are therefore under strong cell cycle control.

This tight control is enabled by the cell cycle kinase Cdk1 and the metabolic regulator cAMP-dependent protein kinase (PKA). These two kinases phosphorylate and thus activate the neutral trehalase Nth1, the enzyme responsible for hydrolyzing trehalose into two glucose molecules (Ewald et al., 2016; Zhao et al., 2016; Solaki and Ewald, 2018). In contrast to trehalases from prokaryotic and higher eukaryotic organisms, yeast neutral trehalase Nth1 is regulated through a unique mechanism involving phosphorylation, calcium binding and association with Bmh proteins (Wera et al., 1999; Panni et al., 2008; Schepers et al., 2012; Veisova et al., 2012).

The N-terminal segment of Nth1 contains five sites, of which four are phosphorylated by PKA (Ser^20^, Ser^21^, Ser^60^ and Ser^83^) and one by Cdk1 (Ser^66^). Ser^20^ and Ser^83^ are constitutively phosphorylated, whereas Ser^21^ and Ser^60^ are phosphorylated to a lesser extent. Ser^60^ and Ser^83^ phosphorylation creates Bmh-binding motifs. However, under nutrient-poor conditions, with low PKA activity, Ser^60^ is not sufficiently phosphorylated; instead Nth1 activity is also regulated by Ser^66^ phosphorylation by Cdk1. This modification likely creates an alternative Bmh-binding motif (Dengler et al., 2021). Therefore, Cdk1 phosphorylates and increases Nth1 activity only when PKA activity is low.

Structural analysis of yeast Nth1 alone and in a complex with Bmh1 revealed that the formation of this complex establishes a suitable spatial arrangement of the catalytic and calcium-binding domains of Nth1by stabilizing the flexible portion of the catalytic domain (Figure 1C) and triggering Nth1 activity (Macakova et al., 2013; Kopecka et al., 2014; Alblova et al., 2017). The calcium ion bound to the calcium-binding domain also stabilizes the interaction between this domain and the flexible portion of the catalytic domain, thus further increasing trehalase activity.

In the plant pathogen *Fusarium graminearum*, the interaction between Nth1 and Bmh1 is enhanced by validamycin A, albeit through an unclear mechanism (Ren et al., 2022). Nevertheless, Nth1 activation by Bmh proteins is a classic example of a mode of action whereby 14-3-3 proteins modulate the enzymatic activity of the bound protein by changing the structure of its active site (Obsilova et al., 2014). In this case, though, Bmh1 binds to Nth1 far from the active center, so this allosteric regulation results from an induced conformational change.

Trehalose also enhances autophagy, thereby mediating neuroprotection in various animal models of Alzheimer´s and Parkinson´s disease (Sarkar et al., 2007; Castillo et al., 2013). When yeast cells face stress, one of the yeast enzymes that catalyze trehalose synthesis is trehalose-6-phosphate phosphatase (Tps2), which dephosphorylates trehalose-6-phosphate to trehalose (De Virgilio et al., 1993). Tps2 positively regulates autophagy in *S. cerevisiae* through processes involving Bmh proteins (Kim et al., 2021). Under nutrient-rich conditions, the Tor protein kinase complex 1 (TORC1) phosphorylates Rim15 kinase, thus inducing its interaction with Bmh1/2 and inactivation. Under nitrogen starvation, Rim15 is dephosphorylated by Tps2, leading to its dissociation from Bmh1/2 and translocation to the nucleus (Pedruzzi et al., 2003).

### 3.3 Spatiotemporal control of meiosis and aggregate homeostasis by 14-3-3 proteins

In meiosis, diploid cells divide into four daughter cells, each of which with half the number of chromosomes of the parent cell. As such, meiosis is a key process that produces haploid gametes for sexual reproduction through two rounds of cell division involving DNA replication (meiosis I) and subsequent segregation of the two chromosomes (meiosis II). Despite the importance of this process, only a few genes involved in meiosis have been identified so far (Tsuchiya et al., 2014; Ballew and Lacefield, 2019), including six novel regulators of meiosis commitment in budding yeast (Gavade et al., 2022), namely nutrient sensing regulator Bcy1, CDK-like kinase Ime2, Polo kinase Cdc5, RNA-binding protein Pes4 and Bmh1/2 (Gavade et al., 2022). Even more recently, two other genes have been implicated in this process–PKA and phosphatase Cdc14 (Zhang et al., 2023). Time will tell whether the limited number of genes associated with yeast meiosis reflects functional redundancy, species-specific differences or lack of knowledge on mechanisms of post-transcriptional regulation.

In *S. cerevisiae*, meiotic gene expression is tightly controlled by RNA-binding proteins, such as Rim4, a major suppressor of the translation of meiotic transcripts (Figure 2C) (Enyenihi and Saunders, 2003; Carpenter et al., 2018). During meiosis, the spatiotemporal regulation of the Rim4 ribonucleoprotein complex is controlled by PKA phosphorylation, yeast 14-3-3 protein binding and dephosphorylation by Cdc14 (Zhang et al., 2023). Accordingly, phosphorylated Rim4 forms a complex with the Bmh1/Bmh2 heterodimer, thereby preventing interactions between Rim4 and mRNA. Within the RNA-binding region of Rim4, two sites (Thr^216^ and Ser^525^) were identified as Bmh-binding motifs after phosphorylation by PKA. These are the primary motifs for robust Rim4-Bmh1/2 interactions during prophase I, when PKA-mediated phosphorylation of Rim4 peaks.

The conserved phosphatase Cdc14 upregulates meiosis-specific autophagy (Feng et al., 2022). Prior to meiotic division, Cdc14 mainly promotes the formation of the Rim4-mRNA complex to its nuclear localization. During meiotic anaphase, increased PKA activity facilitates Rim4 release from bound mRNA, which enables the formation of the Rim4-Bmh1/2 complex and subsequent Cdc14 upregulation and translocation from the nucleus to the cytoplasm. As a result, the Rim4-Bmh1/2 complex breaks down during anaphase.

In the cytoplasm, Cdc14 functions in opposition to PKA, removing Bmh1/2 from Rim4. This dissociation determines the timing of Rim4 degradation by autophagy due to the loss of protection by Bmh1/2 binding and of the translation of Rim4-sequestered mRNAs (Zhang et al., 2023). By regulating the interaction between Rim4 and Bmh1/2, and thus the formation of the Rim4-mRNA complex, spatiotemporally controlled Rim4 phosphorylation by PKA and dephosphorylation by Cdc14 phosphatase determine the distribution, function and stability of Rim4.

Some human and yeast RNA-binding proteins contain prion-like domains rich in Asn and Glu residues that form amyloid aggregates (King et al., 2012). For example, Rim4 has a prion domain and forms amyloid-like aggregates (Berchowitz et al., 2015) to which yeast 14-3-3 proteins bind during meiosis, facilitating Rim4 phosphorylation by the meiosis-specific kinase Ime2 (Herod et al., 2022). This phosphorylation subsequently mediates the disassembly of Rim4 aggregates and contributes to overall protein homeostasis and cellular protection against pathological protein aggregates (Figure 2D). In addition to potentially protecting cells against pathological protein aggregation and age-related diseases, 14-3-3 proteins may also play a key role in Rim4 biology because Bmh proteins contain polyQ stretches and the prion domains of Rim4 are also highly enriched in Glu and Asn residues. Therefore, Bmh proteins might have co-evolved with Rim4 to regulate aggregate disassembly.

Another protein involved in yeast meiosis is the RNA-binding protein Pes4, which regulates the timing and translation of several mRNAs during meiosis II progression after activation by Polo kinase (Esposito and Esposito, 1974; Tsuchiya et al., 2014; Jin et al., 2017). In this meiotic commitment model, Ime2 kinase phosphorylates and activates the middle meiosis transcription factor Ndt80 at the end of prophase I (Gavade et al., 2022). Ndt80 subsequently induces Cdc5, Pas4 and Ime2 gene expression. Bmh1 then directly interacts with Ndt80, Cdc5 and Pes4, stabilizing and maintaining Ndt80 levels, increasing Cdc5 kinase activity and modulating Pes4 to maintain meiotic commitment. In short, Bmh proteins are required to maintain normal Ndt80 levels, to activate Polo kinase and to interact with Pes4, which in turn regulates the timing of translation of several mRNAs important for meiosis II (Gavade et al., 2022). Moreover, these findings support a model in which Bmh proteins, alongside other proteins, control meiotic commitment by protecting cells from meiosis upon nutrient addition.

### 3.4 14-3-3 proteins regulate yeast ion transporters Trk1 and Nha1

Another yeast process involving interactions with Bmh proteins is ion transport across the plasma membrane. In *S. cerevisiae*, for instance, intracellular cation and pH homeostasis are mediated by plasma membrane transporters, such as H^+^-ATPase, K^+^ uptake uniporter, K^+^ efflux channel, Na^+^(K^+^)/H^+^ antiporter and Na^+^(K^+^)-ATPase (Arino et al., 2010, 2019). Potassium storage is mediated by two independent transporters, Trk1 and Trk2 (Madrid et al., 1998). K^+^ is also actively exported from cells by the antiporter Nha1 and the ATPase Ena1, which concurrently export toxic sodium ions. Both Ena1 and Nha1 export four alkali metal cations (Na^+^, K^+^ and their analogs Rb^+^ and Li^+^), but only Ena1 uses ATP hydrolysis as an energy source. Nha1 uses, instead, the input H^+^ gradient generated by the H^+^-ATPase Pma1 (Kinclova et al., 2001; Ruiz and Arino, 2007).

14-3-3 proteins regulate several plasma-membrane ion transporters, including the Na^+^/H^+^ exchanger and the *Arabidopsis thaliana* potassium channel Tpk1 (Lehoux et al., 2001; Latz et al., 2007). In *S. cerevisiae*, the interaction between Bmh1 and Nha1 was validated at the protein level by bimolecular fluorescence complementation, which showed that this interaction enhances cell survival under salt stress (Zahradka et al., 2012). In subsequent *in vivo* and *in vitro* experiments, the phenotypes resulting from disruption of the interaction between Nha1 and Bmh1 were described, whereas biophysical characterization of the C-terminal portion of Nha1 revealed that Bmh binds to phosphorylated Ser^481^ and, to a lesser extent, to Ser^479^ motifs. Upon Bmh binding, the C-terminus of Nha1 transitions from a disordered to an ordered state. Because mutating Ser^481^ to Ala increases cation efflux activity, Bmh binding is likely necessary for Nha1 inhibition, which should be low under standard growth conditions when yeast need to accumulate high levels of K^+^ (Figure 2E) (Smidova et al., 2019).

Another transporter fully or partly regulated by phosphorylation through Bmh binding is the potassium transporter Trk1. However, unlike the aforementioned negative regulation of Nha1, Bmh binding activates Trk1. At low K^+^ levels, Trk1 activity depends on two residues of the second intracellular loop (Ser^882^ and Thr^900^), but Thr^900^ phosphorylation alone is responsible for Bmh binding, in line with the finding that the Thr^900^-to-Ala mutation markedly slows growth under low K^+^ (Masaryk et al., 2023). Yet again, though, the precise molecular mechanisms whereby Bmh proteins modulate the function of Nha1 and Trk1 channels remain unknown.

### 3.5 Endocytosis regulation and crosstalk between ubiquitination and phosphorylation mediated by 14-3-3 proteins

Without endocytosis, yeast cells cannot adapt to environmental changes by inhibiting intracellular signaling, thus downregulating plasma membrane receptors (Horak, 2013). In *S. cerevisiae*, the endocytosis of transporters is mediated by their ubiquitylation by the ubiquitin ligase Rsp5 and its arrestin-related trafficking adaptor (Art) proteins (Polo and Di Fiore, 2008; Becuwe et al., 2012a; MacGurn et al., 2012; Merhi and Andre, 2012). The endocytic activity of Rsp5 adaptor proteins is modulated by phosphorylation and subsequent Bmh binding, as shown in several studies.

The arrestin Rod1 (Art4), for example, is part of the glucose signaling pathway involving the kinase Snf1 and the phosphatase Reg1-Glc7/PP1. In the presence of glucose, Rod1 is activated, but when yeast cells are grown in lactate medium, Rod1 is phosphorylated at Ser^447^ by Snf1 and kept in an inhibited state, sequestered by Bmh proteins (Figure 2F) (Shinoda and Kikuchi, 2007; Alvaro et al., 2016). In other words, phosphorylation prevents Rod1 ubiquitination and hence its activation.

In addition to activating Rod1, glucose also induces endocytosis of the lactate transporter Jen1, associated with its dephosphorylation by Glc7/PP1, and subsequent Jen1 ubiquitination by Rsp5 (Paiva et al., 2002; Becuwe et al., 2012b). Upon endocytosis or glucose depletion, Rod1 is again phosphorylated and bound to Bmh, leading to Rod1 dissociation from the trans-Golgi network, its relocalization to the cytosol and Jen1 recycling to the cell membrane (Becuwe and Leon, 2014). Thus, nutrient availability intrinsincally modulates the plasma membrane expression of nutrient transporters primarily by controlling their ubiquitination and subsequent endocytosis.

Rod1 also regulates the endocytosis of the glucose transporters Hxt1, Hxt3 and Hxt6 through a similar mechanism (via Snf1/Bmh). In addition to Rod1, other Art proteins that regulate Rsp5-dependent transporter downregulation, such as Rog3 (Art7), also bind to Bmh. These interactions are modulated by the carbon source (Andoh et al., 2002; Llopis-Torregrosa et al., 2016).

Another study has shown that the Art protein Csr2 (Art8) is regulated similarly to Rod1 and triggers high-affinity glucose endocytosis (Hovsepian et al., 2017). Csr2 is activated by ubiquitination, whereas glucose addition transcriptionally represses Csr2 to its inactive, deubiquitinated form. Moreover, glucose-induced Csr2 deubiquitination correlates with its association with Bmh in a PKA phosphorylation-dependent manner (Figure 2F).

Bul1 and Bul2 are two other Rsp5 adaptor proteins that interact with Bmh after their phosphorylation by the kinase Npr1 (Belgareh-Touze et al., 2008). Their interaction with Bmh protects the amino acid permease Gap1 from downregulation under low nitrogen conditions. Under high nitrogen conditions, conversely, Sit4 phosphatase dephosphorylates Bul1 and Bul2, which dissociate from Bmh proteins and are subsequently ubiquitylated, ultimately downregulating Gap1 (MacGurn et al., 2011; Merhi and Andre, 2012).

### 3.6 Dynamic regulation of mitochondrial retrograde signaling

Mitochondrial retrograde (RTG) signaling is known in all eukaryotes, from yeast to humans, but its molecular mechanisms are highly diverse (Butow and Avadhani, 2004). *S. cerevisiae* was the first organism in which the RTG pathway was investigated because mitochondrial DNA is not required for its growth. The retrograde reaction is an adaptation of cellular metabolism to disrupted mitochondrial function (Trendeleva and Zvyagilskaya, 2018). Cells with dysfunctional mitochondria experience three main problems, namely glutamate deficiency, energy and adenosine triphosphate (ATP) deficiency, and increased levels of reactive oxygen species (Bui and Labedzka-Dmoch, 2023).

In yeast, RTG signaling is mediated by three transcription factors (Rtg1-3) (Liu and Butow, 2006). As long as mitochondria are functional, the RTG pathway remains switched off, phosphorylated Rtg3 stays bound to Rtg1, and their complex resides in the cytoplasm (Figure 2G). But when mitochondria are damaged, Rtg2 activates the Rtg1/3 complex, either directly through the histone acetyltransferase complex or indirectly through interaction with Mks1, a transcription regulator and a key negative modulator of the RTG pathway (Chiu et al., 2022). So under conditions of lower ATP levels, the RTG pathway becomes active due to Mks1 sequestration by Rtg2, which functions as a cytoplasmic sensor of this pathway (Zhang et al., 2013).

This Rtg2-Mks1 interaction also protects Mks1 from hyperphosphorylation and binding to Bmh1/2 proteins. In turn, Bmh1/2 binding prevents Mks1 degradation by the Skp, Cullin, F-box-containing (SCF) E3 ubiquitin ligase complex. When the RTG pathway becomes inactive again, Mks1 detaches from Rtg2 and binds to Bmh1/2, thereby inhibiting the transfer of the Rtg1/3 complex to the nucleus. Free Mks1 is then degraded by glucose repression resistant protein 1 (Grr1) of the SCF E3 ubiquitin ligase complex (Liu et al., 2005). Mks1 degradation ensures an efficient transition between Rtg2-Mks1 and Bmh1/2-Mks1 complexes. The RTG pathway is also inhibited by Lst8, a seven WD40-repeat protein required for targeting amino acid permeases to the plasma membrane in response to nutrient sensing through TOR signaling (Liu et al., 2001; Rios-Anjos et al., 2017; Bui and Labedzka-Dmoch, 2023).

### 3.7 Regulation of rapamycin-mediated cell signaling and nitrogen catabolite repression

Rapamycin is a small molecule that forms a complex with Fpr1 and Tor (target of rapamycin), thereby blocking their activity in TOR signaling pathway. In *S. cerevisiae*, TOR signaling programmed cell growth in response to nutrients, such as nitrogen and carbon (Beck and Hall, 1999). Treating yeast with rapamycin triggers a rapid and robust starvation response, including changes in the expression of many genes.

In addition to various other roles in cells, yeast 14-3-3 proteins are also involved in rapamycin-mediated cell signaling by binding to the transcription factors Msn2, Msn4 and Rtg3 and their sequestration in the cytoplasm (Bertram et al., 1998). Upon rapamycin treatment, these transcription factors are released and transferred to the nucleus (Beck and Hall, 1999; van Heusden and Steensma, 2001). Therefore, by controlling the subcellular localization of aforementioned transcription factors in the cytoplasm with Bmh, the TOR signaling pathway regulates nutrient metabolism. Moreover, overexpressing both *BMH* genes represses the inhibitory effects of rapamycin, whereas deleting *BMH* genes increases the sensitivity of cells to rapamycin (Bertram et al., 1998).

Bmh1 and Bmh2 regulate rapamycin-mediated transcription differently. Both Bmh proteins are required for rapamycin-induced regulation of different but overlapping sets of genes as they associate with the promoters of at least some of these genes. However, only *BMH2* suppresses genes implicated in ribosome biogenesis and blocks the activation of genes sensitive to nitrogen catabolite repression (NCR) (Trembley et al., 2014). The ability of *S. cerevisiae* to prioritize high-quality nitrogen sources over poor sources is based on the regulation of the nitrogen-responsive transcriptional activators Gln3 and Gat1 of the GATA family (Kulkarni et al., 2006).

Under high-nitrogen conditions, GATA factors are found in the cytoplasm and NCR-sensitive transcription is minimal. Conversely, when nitrogen levels are low, Gln3 is translocated into the nucleus, markedly increasing GATA factor-mediated transcription (Figure 2H). This regulation is based on TORC1-mediate control of Gln3 as rapamycin treatment induces Gat1 and Gln3 translocation into the nucleus, activating their transcriptional activity (Trembley et al., 2014).

Both Gat1 and Gln3 interact with Bmh1/2 proteins *in vivo*, suggesting their role in NCR regulation (Kakiuchi et al., 2007). But the subcellular localization of Gln3 is also regulated by the general amino acid control (GAAC) pathway mediated by the protein kinase Gcn2 (Tate et al., 2017). Together with the transcription factor Gcn4 and Bmh1/2, the GAAC pathway is required for the NCR-sensitive nuclear localization of Gln3 (Hinnebusch, 2005; Tate et al., 2017). Gcn2, Gcn4 or Bmh1/2 loss decreases Gln3 phosphorylation and therefore lowers NCR transcription although exactly how Bmh1/2 proteins control Gln3 localization and function is still unclear.

### 3.8 14-3-3 proteins govern lifespan, apoptosis and heavy-metal resistance

Bmh proteins may also be involved in regulating lifespan, apoptosis and heavy metal resistance. Nutrient-sensitive caloric restriction, TOR and PKA contribute to lifespan extension by enhancing the stress response, protecting cells from the accumulation of age-dependent oxidative damage. As a case in point, Bmh1 phosphorylation at Ser^238^ by PKA increases during chronological aging (Wang et al., 2009). Bmh1 removal then extends lifespan by activating the stress response, presumably by preventing inhibitory Bmh1 effects on these longevity factors, thereby extending lifespan.

Disrupted in many diseases, programmed cell death (PCD) is an important stress-induced process in which 14-3-3 proteins play various regulatory roles (Zhou et al., 2019). For instance, 14-3-3 proteins prevent the human proapoptotic protein Bax from inducing PCD in yeast (Clapp et al., 2012a; Clapp et al., 2012b). In addition, 14-3-3 proteins protect yeast cells against apoptosis induced by rapamycin, leucine deficiency and various stresses, such as cadmium or cycloheximide (Clapp et al., 2012b). Yet, when subjecting normal and PCD-resistant yeast cells to various stresses, cells expressing pro-survival 14-3-3 proteins are more resistant to rapamycin and copper-induced stress, but less so to other stresses such as iron exposure (Eid et al., 2017), suggesting the need for functional vacuoles, which mediate iron and 14-3-3 effects on PCD in yeast cells. In summary, PCD in yeast cells involves a complex interplay between processes that activate and inhibit responses to various stresses via 14-3-3 proteins.

The involvement of Bmh proteins in various signaling pathways has also been demonstrated by recent, large-scale screenings involving yeast collections with one or two deletions. One such study screened targets for improved cadmium tolerance in *S. cerevisiae*, identifying seven target genes, including *BMH1* (Chen et al., 2021). However, cadmium treatment did not induce any significant reduction in *BMH1* mRNA levels. Another study used transposon mutagenesis to investigate mutational tolerance and genome-wide genetic interactions in copy number variant (CNV) strains. These strains not only contribute to evolutionary adaptation but can also have harmful effects and cause CNV-associated diseases such as cancer. In three CNV strains, namely Aneu, ComQuad and Trip1A, a positive genetic interaction was identified between *BMH1* and *GAP1*, and evidence suggests that some CNV strains have altered mitochondrial activity or function, enhanced mitochondrial mutational tolerance, and altered patterns of interactions with *BMH1* (Avecilla et al., 2023). Bmh is a negative regulator of retrograde signaling; therefore, if mitochondria are dysfunctional and retrograde signaling is constitutively activated, mutations in BMH1 can be tolerated. Genetic approaches have also been used in another recent study on the MAPK signaling pathway, which controls fiber growth (Gonzalez et al., 2023). This study proposed the existence of a Ste20p-independent branch (Ste20p is a yeast p21-activated kinase) of this pathway involving adaptor protein Bem4p, whereas the Ste20p-dependent branch requires Bmh1/2 proteins. Moreover, a recent genome-scale screening in *S. cerevisiae* identified *BMH1* among multicopy suppressors of the growth defect caused by glutathione stress (Yasukawa et al., 2023).

## 4 Conclusion

Thanks to recent advances in biochemical, structural, and bioinformatic methods of analysis, we can now better understand the various functions of 14-3-3 proteins, including how 14-3-3 proteins recognize their targets and control their localization and function. 14-3-3 proteins mediate various regulatory functions and are pivotal players in many signaling processes. Nevertheless, several aspects related to 14-3-3 function remain unresolved, particularly structural and functional details about the complexity and modulation of bound targets and the underlying mechanisms of their self-regulation. The main problem remains the lack of available structural data on yeast 14-3-3 protein complexes with full length binding partners. We can only hope that recent advances in cryo-EM methodology will increase the number of available structures of yeast 14-3-3 protein complexes with their full-length binding partners. Since yeast 14-3-3 proteins regulate carbon metabolism, glucose repression, and surface localization of sugar transporters, global protein homeostasis, they may be involved in metabolic diseases associated with sugar metabolism, including diabetes, age-related diseases, including Alzheimer´s disease, Parkinson´s disease and transthyretin amyloidosis as suggested by several studies (Thandavarayan et al., 2008; Watanabe et al., 2008; Wang et al., 2009; Herod et al., 2022) and reviewed in (Kleppe et al., 2011; Knowles et al., 2014; Rial et al., 2023). Further research on the yeast 14-3-3 protein complexes, including structural studies, may therefore help us to gain insights into the role of these key regulators of cellular pathways towards developing effective strategies for targeting and modulating their function.
